# Towards a cure for HIV: a long road ahead

**DOI:** 10.1186/1742-4690-9-S1-I12

**Published:** 2012-05-25

**Authors:** Tae-Wook Chun

**Affiliations:** 1 National Institute of Health, Bethesda, USA

## 

Plasma viremia can be effectively suppressed and maintained below the limits of detection for extended periods of time in most human immunodeficiency virus (HIV)–infected individuals receiving antiretroviral therapy (ART). However, it has not been possible to eradicate HIV by ART alone, likely due in part to the persistence of various viral reservoirs in lymphoid tissues. In this regard, the existence of latently infected, resting CD4^+^ T cells carrying replication-competent HIV has posed one of the major challenges to the long-term control or eradication of HIV in infected individuals on ART. Consequently, there has been considerable focus on therapeutic strategies to reactivate the latent viral reservoir using various agents, such as cytokines, histone deacetylase inhibitors, and mitogens, under the assumption that these cells would die due to HIV induced cytopathic effects and antiretroviral drugs would prevent spread of infection. However, such approaches have shown no clinical benefit to date. Moreover, it also has become clear that HIV persists in subsets of CD4^+^ T cells in blood and lymphoid tissues of infected individuals receiving ART. Recent data from our laboratory will be discussed which will include potential mechanisms of HIV persistence and prospects for eradication and new therapeutic approaches in HIV-infected individuals receiving effective ART.

